# Structure of the CRISPR Interference Complex CSM Reveals Key Similarities with Cascade

**DOI:** 10.1016/j.molcel.2013.08.020

**Published:** 2013-10-10

**Authors:** Christophe Rouillon, Min Zhou, Jing Zhang, Argyris Politis, Victoria Beilsten-Edmands, Giuseppe Cannone, Shirley Graham, Carol V. Robinson, Laura Spagnolo, Malcolm F. White

**Affiliations:** 1 Biomedical Sciences Research Complex, University of St Andrews, Fife KY16 9ST, UK; 2 Institute of Structural Molecular Biology and Centre for Science at Extreme Conditions, University of Edinburgh, Edinburgh EH9 3JR, UK; 3 Department of Chemistry, 12 Mansfield Road, University of Oxford, Oxford OX1 3TA, UK

## Abstract

The Clustered Regularly Interspaced Palindromic Repeats (CRISPR) system is an adaptive immune system in prokaryotes. Interference complexes encoded by CRISPR-associated (*cas*) genes utilize small RNAs for homology-directed detection and subsequent degradation of invading genetic elements, and they have been classified into three main types (I–III). Type III complexes share the Cas10 subunit but are subclassifed as type IIIA (CSM) and type IIIB (CMR), depending on their specificity for DNA or RNA targets, respectively. The role of CSM in limiting the spread of conjugative plasmids in *Staphylococcus epidermidis* was first described in 2008. Here, we report a detailed investigation of the composition and structure of the CSM complex from the archaeon *Sulfolobus solfataricus*, using a combination of electron microscopy, mass spectrometry, and deep sequencing. This reveals a three-dimensional model for the CSM complex that includes a helical component strikingly reminiscent of the backbone structure of the type I (Cascade) family.

## Highlights

• The CSM complex from *Sulfolobus solfataricus* has been purified and characterized
• EM reveals a helical backbone with striking similarities to the Cascade complex
• Mass spectrometry defines the subunit stoichiometry and organization of the complex
• CSM subunits are modified by methylation, acetylation, and phosphorylation

## Graphical Abstract

**Figure:**
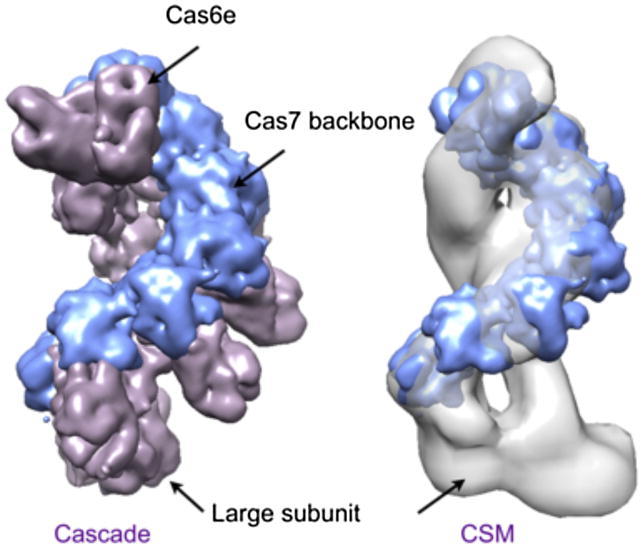

## Introduction

The Clustered Regularly Interspaced Palindromic Repeats (CRISPR) system is a prokaryotic adaptive immune system that targets and degrades invading genetic elements. DNA fragments from mobile elements are captured and incorporated into the host genome at a CRISPR locus, flanked by direct repeat sequences, in a poorly understood process termed “adaptation” (van der Oost et al., 2009; Yosef et al., 2012). Transcription of the locus generates a long pre-CRISPR RNA (pre-crRNA) transcript that is processed into unit-length crRNAs by specific cleavage. Each crRNA is composed of a single “spacer” region homologous to a mobile genetic element, with a variable flanking region derived from the CRISPR sequence that flanks the spacer. crRNAs are loaded into a ribonucleoprotein complex and utilized for homology-dependent targeting and cleavage of cognate mobile elements in a process known as “interference” (Marraffini and Sontheimer, 2008). These complexes have been classified into three major types, I–III, characterized by the presence of a signature CRISPR-associated (Cas) protein: Cas3, Cas9, and Cas10 for types I, II, and III, respectively (Makarova et al., 2011b). In addition, types I and III share a variable number of Repeat Associated Mysterious Protein (RAMP) subunits. The RAMP domain is a derivative of the RNA Recognition Motif (RRM) fold and is often involved in RNA binding and/or cleavage (Makarova et al., 2011a).

The type IIIA complex, also known as the CSM complex, is found in a wide variety of bacteria and archaea. In *Staphylococcus epidermidis*, CSM is encoded in an operon that includes the *csm1-6* genes and has been shown to limit plasmid conjugation by targeting invading DNA for degradation (Marraffini and Sontheimer, 2008). CSM is associated with crRNA generated by cleavage of pre-crRNA by Cas6 and 3′-end processing by an unknown nuclease (Hatoum-Aslan et al., 2011). The CRISPR locus in the host genome is not cleaved by type IIIA systems, as there is a requirement for a mismatch region at the boundary of the repeat-spacer sequence: a condition that is met for foreign DNA targets but not for the genomic locus, where the crRNA matches perfectly to the genomic sequence (Marraffini and Sontheimer, 2010).

Although the type IIIA systems provided the first example of unequivocal DNA targeting by the CRISPR system, there has been little progress in the biochemical characterization of any CSM complex. Here, we report the purification and structural characterization of the CSM complex from the archaeon *Sulfolobus solfataricus*. Electron microscopy (EM) reveals an extended, intertwined helical conformation that suggests a backbone formed by RAMP subunits with striking similarities to that of the type IE Cascade complex (Wiedenheft et al., 2011). Mass spectrometry (MS) was used to define the subunit composition and subcomplex organization. Deep sequencing of the crRNA copurifying with the complex unveils a remarkable specificity for crRNA that suggests a very biased uptake mechanism, perhaps coupled to the Cas6 endonuclease.

## Results

### Identification and Purification of a CSM Complex from *S. solfataricus*

**Figure 1:**
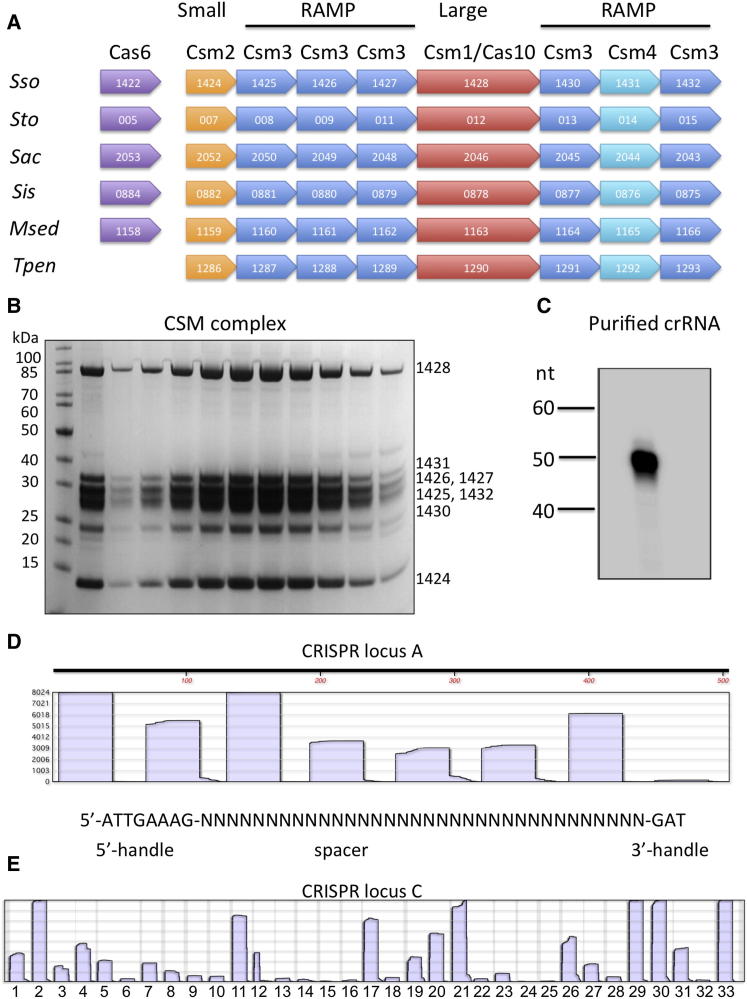
Purification and RNA Content of the CSM Complex from *S. solfataricus* (A) Gene organization of the *csm* locus in selected crenarchaeal species. The gene order is conserved and typically includes a gene encoding Cas6 for crRNA processing. Gene numbers are shown and are contiguous on the genome. Abbreviations are as follows: Sso, *S. solfataricus*; Sto, *S. tokodaii*; Sac, *S. acidocaldarius*; Sis, *S. islandicus* strain M.14.25; Msed, *Metallosphaera sedula*; Tpen, *Thermofilum pendens*. *T. pendens* Cas6 is present elsewhere on the genome. (B) Fractions of the CSM complex eluting from the final gel-filtration column during purification. All eight subunits can be visualized and detected by MS. (C) RNA purified from the purified CSM complex. A single discrete band around 50 nt was observed. (D) A linear coverage map for a series of eight spacers from the *S. solfataricus* P1 A locus is shown. 5′ ends were defined by the 8 nt 5′-handle derived from cleavage of the repeat by Cas6. (E) Linear coverage map for the entire CRISPR C locus from *S. solfataricus* P1, highlighting the variability in coverage.

The organization of *csm* loci in a selection of crenarchaea (Figure 1A) shows conservation of gene order across these species, with eight *csm* genes typically preceded by a *cas6* gene for crRNA processing. In addition to RAMP-domain subunits, partially conserved “large” and “small” subunits have been identified in most type I and III interference complexes (Makarova et al., 2011a). The gene encoding the large subunit Csm1 is annotated as a split gene encompassing *sso1428* and *sso1429* in the *S. solfataricus* genome sequence (She et al., 2001) but was found intact in the current study and named *sso1428*. The small subunit, Sso1424, probably corresponds to Csm2 in *S. epidermidis*, although sequence similarity is very limited. There are six *csm* genes encoding RAMP domain proteins (Figure 1A), compared to three in *S. epidermidis* (*csm3–csm5*) (Marraffini and Sontheimer, 2008). Previously, Sso1431 was predicted to be a member of the Csm4 family (Makarova et al., 2011a), and sequence comparison using HHPRED (Söding et al., 2005) reveals a clear structural match to the *P. furiosus* Cmr3 subunit of the type IIIB CMR complex for RNA targeting (Shao et al., 2013), in agreement with the prediction that both Csm4 and Cmr3 are members of the Cas5 subfamily of RAMP proteins (Makarova et al., 2011a). Of the other RAMP subunits, Sso1426 has the closest predicted structural match to the Cas7 family (Lintner et al., 2011), which is thought to make up the crRNA binding helical backbone of all the type I complexes (Makarova et al., 2011a; van Duijn et al., 2012). We therefore assign this subunit to the Csm3 family as suggested previously (Makarova et al., 2011a). Other RAMP subunits are considered as Csm3 paralogs in Figure 1.

We purified the CSM complex from *S. solfataricus* by employing an approach previously used to isolate the type IIIB (CMR) complex (Zhang et al., 2012). This involved expression of one tagged subunit of the complex, in this case either the *sso1428* or *sso1431* gene encoding Csm1 or Csm4, respectively, from a viral expression vector, followed by a combination of affinity, ion exchange, and gel filtration chromatography. The complex was purified as a homogeneous population eluting as a single peak in the final chromatography step, as confirmed by SDS-PAGE analysis (Figure 1B). The presence of all eight subunits was confirmed by MS. None of the Cas6 paralogs present in *S. solfataricus* copurified with the complex, suggesting that Cas6 is not stably associated.

### Sequence Analysis of RNA Copurifying with CSM

The RNA copurifying with the CSM complex was isolated, end labeled, and analyzed by denaturing gel electrophoresis (Figure 1C). The RNA, which was remarkably defined in size at around 50 nt, was cloned and deep sequenced on an Illumina platform. From the 5.77 million reads of 36 nt obtained after filtering, 5.45 million (94%) could be mapped to the six CRISPR loci present in the *S. solfataricus* P1 strain from which the complex was purified (Lillestøl et al., 2006), suggesting highly specific uptake of crRNA by the CSM complex. The six CRISPR loci in *S. solfataricus* are designated with the letters A–F and are characterized by two different types of repeat sequence, the A and B repeats being significantly different from those of C, D, E, and F (Lillestøl et al., 2006). CSM-derived crRNAs from the A and B loci made up 89% of the total matches, which together constitute 32% of the total spacers present on the genome. The D, E, and F loci were significantly underrepresented, constituting 11% of the matches, in sharp contrast to the fact that they constitute 68% of the spacers in the genome (Table S1). On the contrary, deep sequencing of the CMR complex crRNA revealed a bias toward the C and D loci (Zhang et al., 2012). These biases may reflect functional coupling of the CSM and CMR complexes with different Cas6 paralogs that have complementary specificity for the two CRISPR repeat families present in *S. solfataricus*.

Deep sequencing revealed that, as observed previously for the crRNA component of the CMR complex (Zhang et al., 2012), crRNA begins with the repeat-derived 8 nt 5′ handle (Figure 1D). Spacers in *S. solfataricus* are quite variable in length, ranging from 34 to 48 nt with a median value around 39 nt (Lintner et al., 2011). Thus, in CSM, the “average” spacer of 39 nt will be bounded by 8 nt of repeat-derived 5′ handle and around 3 nt of repeat-derived 3′ handle (Figure 1D). The secondary cleavage of crRNA in this case may occur after binding to CSM, with the complex defining the final length of the crRNA. As observed previously for the crRNA from the CMR complex, there is considerable variation in the coverage of individual spacers in the sequencing data. For example, in locus C, spacers 2, 11, 17, 21, 29, 30, and 33 are highly represented whereas other spacers are represented at much lower levels (Figure 1E). There is no general trend toward higher coverage at the 5′ end of the array, which might be explained by higher levels of transcription of spacers nearer the promoter, as has been observed for *Pyrococcus furiosus* (Hale et al., 2012). The reasons for the variability observed may be a combination of differences in expression due to the presence of internal promoters in captured spacers, differences in the efficiency of processing by Cas6 due to spacer sequence or structure effects, or variability in the cloning efficiency.

### Electron Microscopy

**Figure 2:**
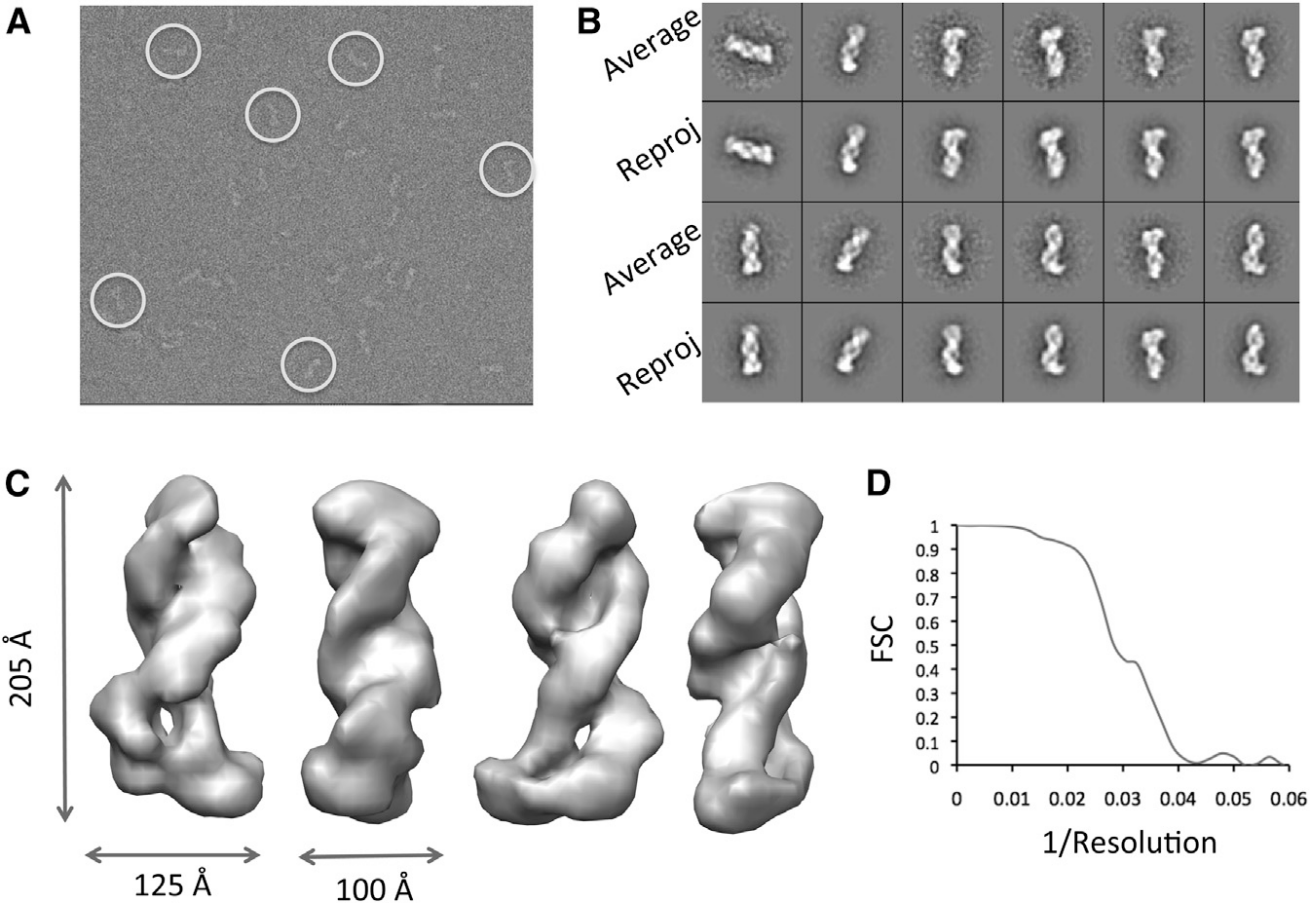
3D-EM Reconstruction of the CSM Complex (A) Raw micrograph, with representative single particles in white circles. (B) Class averages and reprojections from the 3D reconstruction. (C) Surface representation of the full 3D CSM volume. (D) FSC plot.

To gain insights into the assembly of the CSM complex, we performed EM coupled to single-particle analysis. Individual images of the complex showed an elongated shape. Image classification allowed a first appreciation of a coiled structure, where two filaments are intertwined. Most particles fell on the EM grids on the long axis, in side or tilted views. Top views were, however, not included in the reconstruction because they might have been poorly stained as a result of the overall length of the complex. Three-dimensional (3D) reconstruction and analysis of CSM confirmed these initial observations, revealing an assembly formed by two intertwined protein filaments, one thicker than the other, connected by a wider base (Figure 2). The overall dimensions of the complex are 205×125×100 Å. The resolution of the final reconstruction was determined as ∼30 Å, calculated by Fourier shell correlation with a 0.5 cutoff.

### Subunit Composition Probed by MS

In order to investigate the composition of the CSM complex, we carried out MS analysis. The complex purified with a 10× His-tag attached to the C terminus of the subunit Sso1428 or Sso1431 was first analyzed by denaturing high-performance liquid chromatography–mass spectrometry (HPLC-MS), which confirmed the presence of all eight subunits (Table S2). The RNA component was characterized by phenol extraction of the CSM complex followed by ethanol precipitation (Hernández et al., 2009). An MS spectrum showed a single charge-state series with a mass measured as 16,520 Da, consistent with the 50 nt crRNA (assuming an average mass of 321.5 Da for the four major ribonucleotide residues). The unusual broadness of the charge-state peaks (Figure S1) most likely reflects the sequence heterogeneity of the crRNA. In addition, proteomics experiments identified a series of posttranslational modifications (PTM) in CSM subunits (Table S2). The most prominent PTM was methylation, present in all eight subunits. Extensive methylation of lysine residues in crenarchaea has been reported previously and is suggested to be an adaptation conferring enhanced protein thermostability (Botting et al., 2010). The small subunit (Sso1424) was found to be 15 amino acid residues shorter than the annotated sequence, beginning with an acetylated N-terminal Ser-16 and including a total of seven methylated lysines. Subunits Sso1425 and Sso1431 were also found to be phosphorylated. Recently, over 500 phosphoproteins from *S. solfataricus* have been identified, although the role of phosphorylation in this organism is not well understood (Esser et al., 2012). The measured masses of the Sso1426 and Sso1427 subunits were within 70 Da of one another (Table S2), precluding the possibility of discriminating between them in the MS experiments.

**Figure 3:**
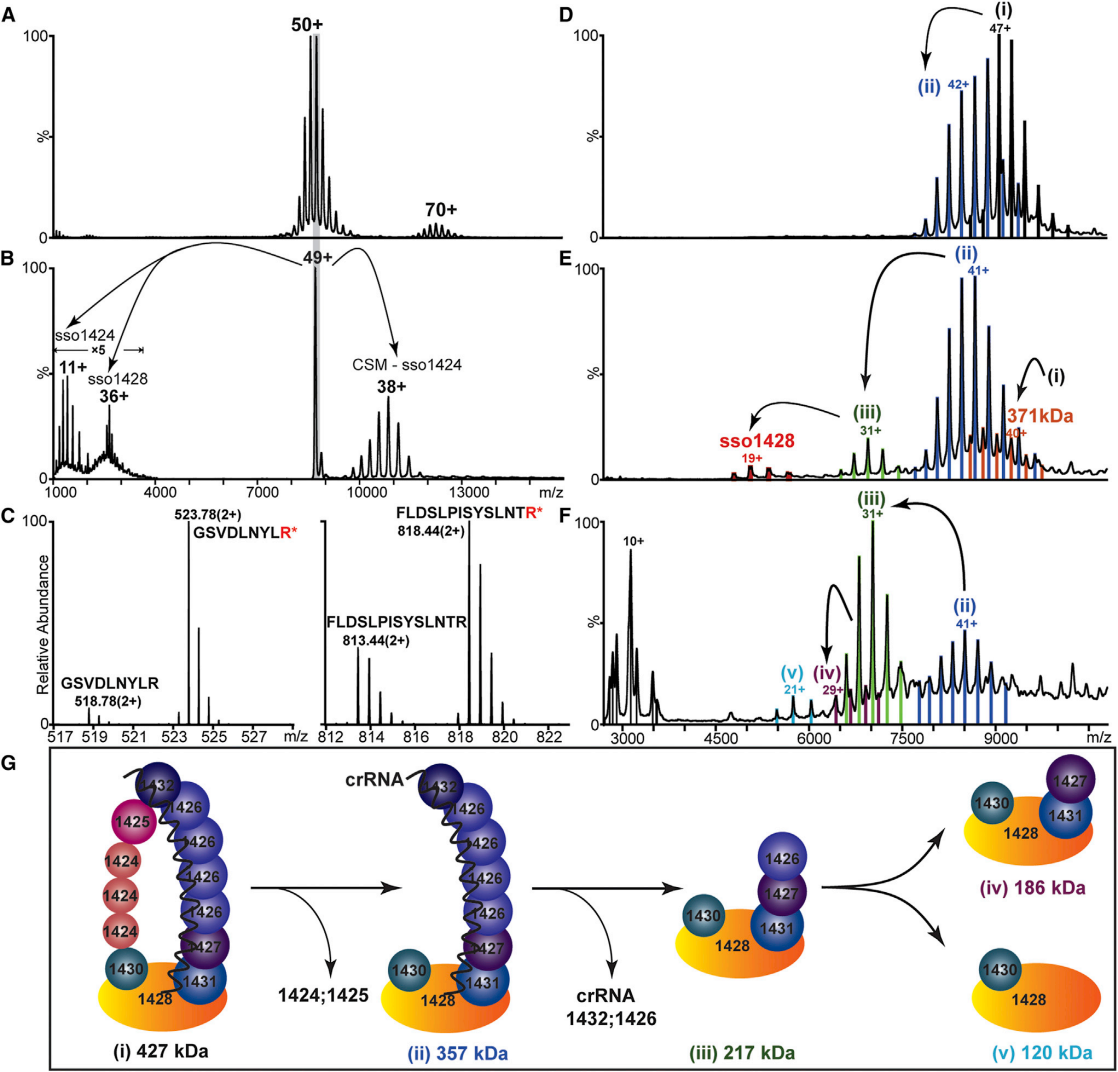
MS Analysis of the CSM Complex Establishing Its Composition, Subunit Connectivity, and crRNA Binding (A) MS spectrum of the intact CSM reveals a well-resolved charge-state series at 8,500 m/*z* with a molecular mass of 427,789 Da, 122 kDa higher than the expected mass for a stoichiometric complex comprising eight subunits and one crRNA. (B) The 49+ charge state of the complex was selected and subjected to acceleration, and dissociation of subunits Sso1424, Sso1428, and Sso1426/7 was observed by tandem MS. (C) The molar ratio of Sso1426:Sso1428 was determined as 4:1 by relative quantification of tryptic peptides of Sso1426 and Sso1428 (GSVDLNYLR and FLDSLPISYSLNTR, respectively; see Table 1 and Table S3). Labeled peptides of the same sequences were synthesized and used as reference. (^15^N,^13^C)-labeled residues are colored red. (D–F) Disassembly of the CSM complex resulted in a series of subcomplexes (i–v) in solutions of decreasing pH: 3.9 (D), 3.5 (E), and 3.2 (F). (G) A complete CSM subunit interaction map was derived from MS data, including intact subcomplexes, crosslinking, and quantitative analysis (see also Figures S1–S3 and Tables S2–S6). The crRNA binds to subunits making up the major backbone and dissociates together with three copies of Sso1426 and Sso1432.

With the masses of the protein and RNA components established experimentally, we then recorded a MS spectrum for the intact complex. MS spectra for CSM preparations with a His-tag attached to either Sso1428 or Sso1431 were recorded under nondenaturing conditions. Spectra for both preparations were very similar, dominated by a single, well-resolved charge-state series at around 8,500 m/*z* (Figure 3A). The masses of the intact complexes tagged on Sso1431 and Sso1428 were measured as 427.7 and 427.6 kDa, respectively (Figure S2), indicating a stoichiometric existence for these subunits in the complex. Under the conditions employed, some dimers (855 kDa) of low intensity were observed, presumably due to the multiple occupancy of the complex within the final offspring droplets, which is an artifact of the electrospray process (Lane et al., 2009). Gas-phase dissociation of Sso1424, Sso1428, and Sso1426/7 was observed upon tandem MS (Figure 3B). These data suggest that the CSM complex exists as a homogeneous population comprising one single crRNA and eight distinct protein subunits, of which Sso1428 and Sso1431 are present in equimolar quantities.

The measured mass for the intact complex was 122 kDa higher than the sum of the masses of its constituent subunits and crRNA, suggesting that some subunits of CSM existed in multiple copies. To determine the subunit stoichiometry, we turned to quantitative proteomics, using a labeling approach. We selected representative tryptic peptides from each subunit for isotopic labeling at C-terminal R/K residues, and to ensure a 1:1 molar ratio the peptide from the largest subunit, we conjugated Sso1428 with the remaining peptides, resulting in eight dipeptides for synthesis (two for the subunit Sso1430; Table S3). Each synthetic dipeptide was individually spiked into the CSM preparation before trypsin digestion, and the resultant peptide mixtures were analyzed by liquid chromatography–mass spectrometry (LC-MS). Comparison of signals generated by the labeled peptides resulted in a list of ratios of Sso1428 relative to the other seven CSM subunits; uncertainties still existed, however, especially for the subunits Sso1424, Sso1425, and Sso1426 (Table 1). We therefore resorted to MS of the intact complex and performed an exhaustive mass search based on the intact mass measurement (427,611 Da). For this, we allowed flexibility of copy numbers of these three subunits by one, with the stoichiometry of remaining subunits fixed according to Table 1. The search resulted in only one hit within a mass error of 3% and thus unambiguously assigned the relative molar ratios of the eight CSM subunits Sso1424 to Sso1432 to be 3:1:4:1:1:1:1:1, with Sso1424 and Sso1426 present in three and four copies, respectively, and unit stoichiometry for the others (Table 1 and Table S4).

Having established its subunit composition and stoichiometry, we proceeded to investigate the organization of subunits within the intact complex. For this, we employed a combination of crosslinking (CXMS) and in-solution disassembly. The intact complex could be disrupted by decreasing the pH, and a series of subcomplexes sized from 357 kDa down to 120 kDa (Figures 3D–3F, species i–v) were formed. We employed tandem MS to assign the subcomplexes, revealing their compositions, all of which contained the largest subunit Sso1428 (Figure S3, Table S5). This allowed us to distinguish a stable “base” subcomplex comprising single copies of Sso1428, 1430, and 1431 and two copies of 1426 and 1427. Further dissociation of this subcomplex led to the hetero-dimer Sso1428:1430 (120 kDa).

This disassembly pattern allowed us to deduce an interaction map, with assistance from the characteristic EM structure, with an intertwined major and minor filament (Figure 2). Of the 13 CSM subunits, 12 form two filaments stemming from the large one, Sso1428 (Figure 3G). The minor filament (Sso1430–1425) contacts the base subunit via Sso1430 and dissociates first at acid pH. This was followed by loss of subunits Sso1432 and three copies of Sso1426, which constitute the bulk of the major filament. This loss correlated with the loss of the crRNA molecule, suggesting an important role for Sso1426 in crRNA binding. The order of the subunit interactions was further confirmed by chemical crosslinking with a Bis[sulfosuccinimidyl] suberate deuterated and nondeuterated pair to generate crosslinked peptides with a readily distinguishable isotopic signature. Over 100 crosslinks were identified, among which six repeatedly identified intersubunit links were considered (Table S6). These include the large subunit Csm1 (Sso1428) crosslinking with both Sso1430 and Csm4 (Sso1431), which supports the identification of these three subunits at the base of the CSM structure. At the head of the structure, the Sso1425 subunit crosslinked to both Sso1426 and Sso1432. A crosslink between Sso1424 and Sso1427 suggests that the two helical filaments contact one another near the base.

**Figure 4:**
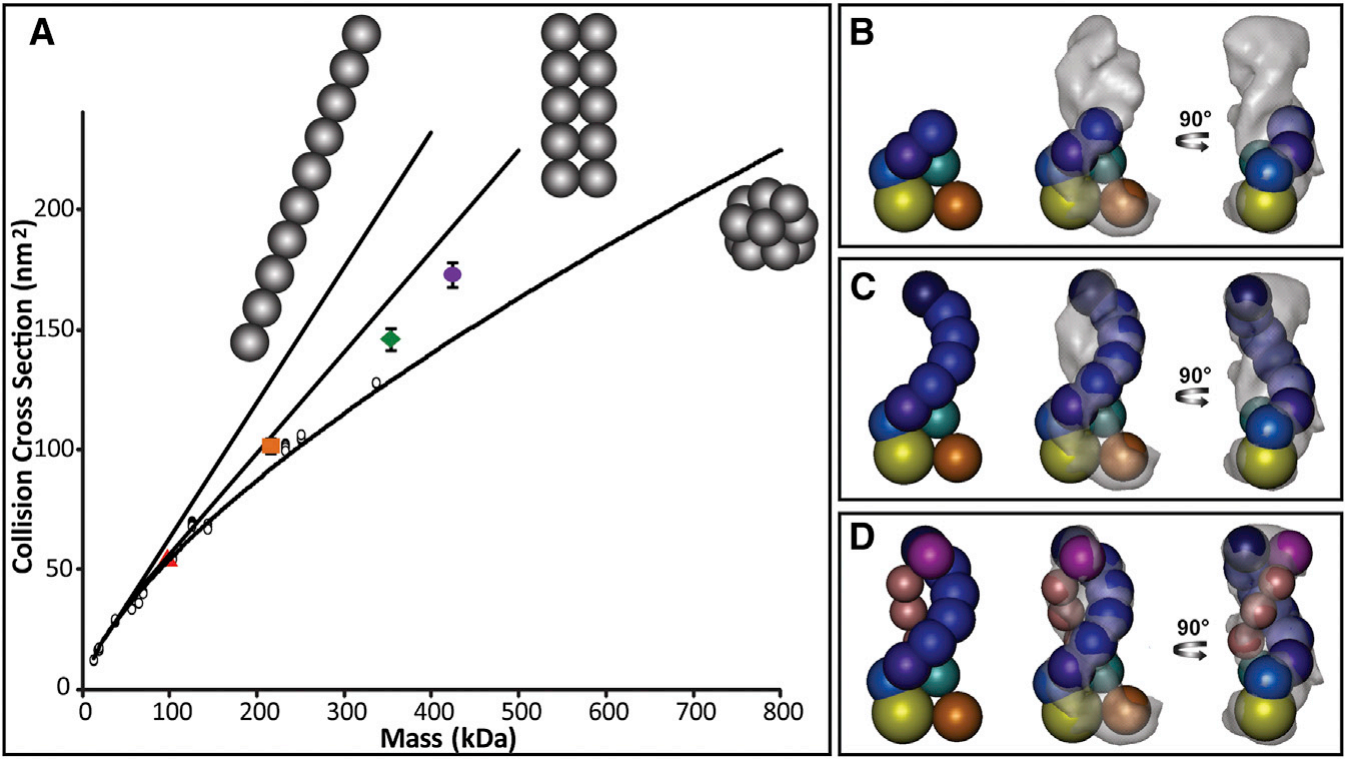
Ion Mobility Measurement of the CSM Complex and Its Subcomplexes (A) CCS values measured for the intact complex (purple circle), the 357 kDa (green diamond) and 216 kDa (orange square) subcomplexes, and the largest subunit Sso1428 (red triangle) are plotted against their masses. Three trendlines are shown for linear, linear dimer, or collapsed “globular” conformations (left to right) for complexes composed of monomers (25 kDa). Considerable deviation from all conformation is evident for the intact complex and the two subcomplexes. (B–D) Coarse-grain structural models, calculated for the intact complexes (D) and the 357 kDa (C) and 216 kDa (B) subcomplexes and fitted into the CSM EM map. Each subunit is represented by a sphere, sized proportionally to its mass, except that the largest Sso1428 is divided into two domains.

To explore the spatial arrangement of the subunits, we used ion mobility MS (IM-MS) to measure the collision cross sections (CCS) for the intact complex and subcomplexes (Figure 4A, Table 2). Experimental CCS values were used as restraints for structural characterization in which candidate models were scored by the closeness of fit between the experimental and calculated CCS values (Alber et al., 2005; Politis et al., 2010). A coarse-grained structural model for the CSM complex was generated this way, which is in good agreement with the EM map (Figures 4B–4D).

### A Model for the CSM Complex Structure and Composition

**Figure 5:**
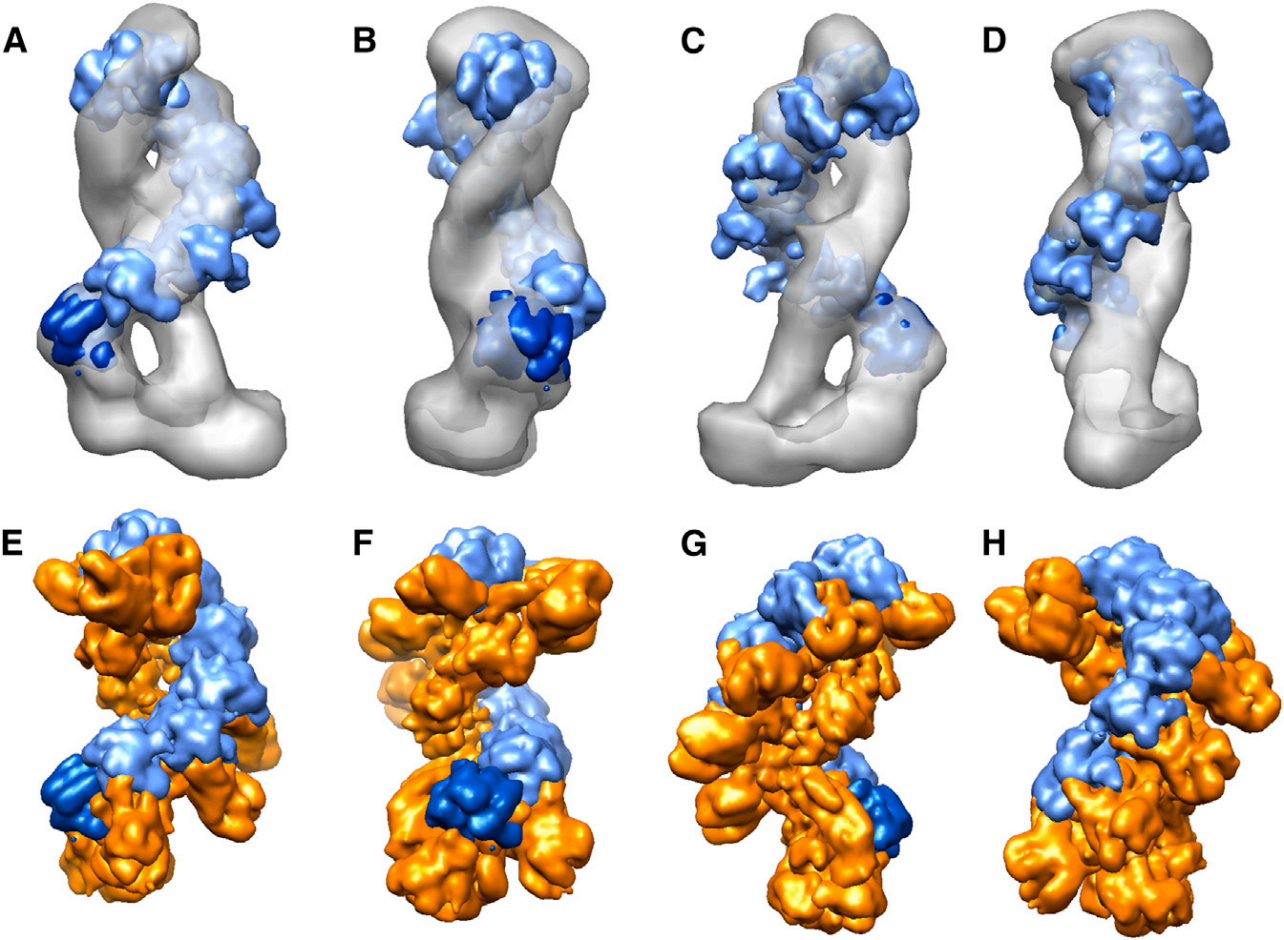
Fitting the Cascade Backbone in CSM and Comparison of the Two Structures (A–D) Orthogonal views of CSM (gray surface) with fitted Cas5 (dark blue) and six Cas7 proximal domains (light blue). (E–H) Orthogonal views of the Cascade complex from *E. coli*, where Cas5 and Cas7 proximal domains have been colored blue for direct comparison with CSM.

The EM map of the CSM complex revealed an elongated structure, formed by two intertwined filaments connected at one end by a wide base (Figures 2 and 5). The level of detail obtained with 3D EM techniques allowed interpretation of the structure with fitting experiments. We built a backbone for the RAMP proteins on the basis of the Cas7 backbone present in the EMD-5314 map for the Cascade complex (Wiedenheft et al., 2011). Cas7 in Cascade is a larger polypeptide in comparison to the RAMP subunits present in CSM; therefore, we used only proximal domains, which are similar to RAMPs in size, to generate a backbone. We built a backbone using six Cas7 proximal domains (shown in light blue in Figure 5) that correspond to RAMP subunits Sso1427, 4 monomers of Sso1426, and Sso1432. At the base of the backbone, the Cas5 subunit from the bacterial Cascade complex (shown in dark blue in Figure 5), corresponding to Csm4 (Sso1431), is shown. This is consistent with volumetric observation, as well as with the CSM stoichiometry determined by MS. The pitch of the CSM backbone is identical to that of Cascade (Figures 5A–5D), whereas the CSM complex is slightly longer than Cascade (205 Å compared to 190 Å). The position of the RNA within this assembly remains elusive to EM at this resolution, but the thicker diameter of the major backbone is consistent with the presence of bound crRNA, and this corresponds to the binding orientation observed in Cascade. The thicker filament is ∼130 Å long, in line with the size of the bound RNA. On both faces of the complex, the crevices between the two filaments (Figures 5A and 5C) have a width of ∼24 Å and a length of ∼130 Å. This is morphologically compatible with the diameter and length of a 38 bp DNA duplex (Figure S4), suggesting a possible role in target recognition at one of these two interfaces. This could also allow strand exchange with the crRNA bound along the Cas7 backbone. Consistent with this possibility, the purified CSM complex binds duplex DNA species with high affinity (K_D_ around 100 nM), although sequence-specific binding could not be demonstrated because of the diversity of the crRNA bound to the complex (Figure S4). The size of the base of the structure is compatible with the expected volume of the full-length Cas10 (large) subunit. It should be noted that Cas10 could not fit within the density of the filaments, both of which are too thin to accommodate it. At the base of the helical backbone, the two structures are not comparable. This is consistent with the distinct structures of the large subunits of the type I and type III complexes, Cse1 and Cas10, respectively (reviewed in Reeks et al., 2013b).

## Discussion

### Comparison with Other CRISPR Interference Complexes

Our data suggest that *S. solfataricus* CSM, and by extension all of the type IIIA complexes, are related structurally to type I complexes, sharing a crRNA-binding helical backbone built from Cas7-family RAMP domain proteins. In this case, the backbone interacts at one end with the Csm1-Csm4 (Cas10-Cas5) base domain, which may bind the 5′ end of the crRNA. This domain probably corresponds to the “crab claw” domain formed by the Cmr2 and Cmr3 subunits of the type IIIB complex (Zhang et al., 2012). Recent structures have shown that these two subunits form a deep crevice at their interface, which ends at the characteristic “cyclase” motif of the Cas10 subunit (Osawa et al., 2013; Shao et al., 2013). The structures reveal binding pockets for two nucleotides, which could represent part of a larger crRNA-binding site (Osawa et al., 2013). The conserved cyclase domain of Cas10 may thus play a role in recognition of the 5′ end of the crRNA rather than functioning as a catalytic domain. Additional biochemical studies are needed for investigation of this possibility.

The bulk of the crRNA-binding backbone is made up of four copies of Sso1426 and one of Sso1427, which can be regarded as Cas7 (or Csm3) family proteins. One end of the backbone is defined by an interaction at the base between the Cas7-like Sso1427 and the Cas5-like Sso1431, analogous to the Cas5-Cas7 core of type I complexes (Makarova et al., 2011b). The backbone is capped at the head by the Sso1432 and Sso1425 subunits, themselves RAMP family proteins, which presumably bind the 3′ end of the crRNA. Unlike the type IE complex, there is no 3′ crRNA hairpin structure and no integral Cas6 subunit. A second helical filament consisting primarily of three copies of the “small” subunit Sso1424 winds back down to link with the foot domain through the Sso1430 subunit. Recently, it has been suggested that the small subunits (Cse2, Cmr5, and Csm2) of all the type I and type III complexes are structurally related (Makarova et al., 2011a), and there are some structural data in support of this (Reeks et al., 2013a). However, there is no detectable sequence similarity between Sso1424 and the Csm2 subunits of CSM complexes from other species such as *S. epidermidis*, let alone Cmr5 or Cse2 family proteins.

The similarity observed between the structures of the type I and type IIIA complexes is perhaps unsurprising given their similar function: both use bound crRNA to detect invading duplex DNA moieties, promoting strand exchange to form an R-loop that is a signal for DNA degradation. In contrast, the EM structure of the type IIIB (CMR) structure appears very different from that of the type IIIA complex, despite the fact that they share much clearer homology than either does with Cascade. The “body” of the CMR complex comprises a number of RAMP domain proteins (Cmr1, Cmr4, Cmr5, and Cmr6) that are assumed to bind RNA. However, they are not obviously arranged in the helical conformation seen for the type I and type IIIA complexes, instead appearing to form a more compact structure (Zhang et al., 2012). This may reflect the fact that CMR targets RNA substrates, which will not have the rigid helical structure of dsDNA. It remains to be seen whether all CMR complexes adopt this compact organization or whether this is specific to the crenarchaeal system.

### crRNA Binding and Processing in Type III Complexes

crRNA in *S. solfataricus* is generated by the cleavage of a primary pre-crRNA transcript within the repeat sequence by the Cas6 endonuclease (Reeks et al., 2013c; Shao and Li, 2013). This generates crRNA with a defined 8 nt repeat-derived 5′ handle, followed by a spacer sequence that can vary from 34 to 44 nt in length (Lintner et al., 2011) and a 3′ repeat-derived handle of 15–16 nt. This primary product is loaded, apparently without further processing, into the type IA complex (Lintner et al., 2011). However, in the type IIIB complex, further maturation was observed as generating shorter crRNAs with reduced 3′ ends (Zhang et al., 2012). In studies of the type IIIA system from *S. epidermidis*, mature crRNA of two sizes (39 and 45 nt) were observed. It has been proposed that crRNA is trimmed at the 3′ end by an unknown nuclease in a process directed by a ruler mechanism measured from the (Cas6-derived) 5′ end (Hatoum-Aslan et al., 2011).

Deep sequencing of the *S. solfataricus* CSM RNA complement confirmed that crRNAs were defined by a common 5′ end resulting from cleavage of the CRISPR repeat by Cas6, as expected. This suggests that, as observed previously for the *S. solfataricus* CMR and *S. epidermidis* CSM complexes, maturation involves 3′-end trimming. The most likely explanation may be that the complexes bind crRNA with an element of recognition of either the 5′ end or the 5′ handle sequence (or both), perhaps in the crevice formed by the Cas10 and Cas5 proteins as described above. Binding of crRNA by Cas7 family proteins results in the protection of a defined length of crRNA, and any excess is trimmed from the 3′ end by a nonspecific 3′-to-5′ exonuclease, as yet unidentified. In support of this, no mass shift was observed for the CSM complex treated with ribonuclease A, suggesting that the mature crRNA is fully protected by the complex (data not shown). The observation of two crRNA lengths differing by 6 nt in *S. epidermidis* CSM and *P. furiosus* CMR could be explained by differences in the number of Cas7-type crRNA-binding subunits present in the backbones of the complexes, as 6 nt approximates to the expected RNA-binding site size of Cas7 (Lintner et al., 2011). In other words, complexes with a 6-RAMP backbone would bind 36 nt of crRNA, while addition of a seventh RAMP subunit would allow the binding of a 42 nt crRNA. By contrast, *S. solfataricus* CSM appears to adopt a single, defined subunit composition with a single length of bound crRNA. It is possible that the control of backbone length by multimerization of RAMP proteins is not always precise.

### Target Degradation by Type IIIA Interference Complexes

The large (Cas10) subunits of the type IIIA and type IIIB complexes, Cmr2 and Csm1, each have an N-terminal HD-nuclease-like domain, reminiscent of that found in the Cas3 helicase-nuclease that is recruited for the degrading of viral DNA by Cascade. It was originally assumed that this would constitute the active site for all the type III complexes. However, this appears not to be the case for the *P. furiosus* CMR complex (Hale et al., 2012), and recent structural comparisons have highlighted the incomplete conservation of HD domains in all the type III complexes (Reeks et al., 2013b). Although CSM binds dsDNA with high affinity, we have so far been unable to demonstrate any crRNA-dependent nuclease activity for the type IIIA complex in vitro (C.R., J.Z., S.G., and M.F.W., unpublished data), and no other publication has reported such an activity, despite the fact that the complex was first reported to target DNA in vivo in 2008 (Marraffini and Sontheimer, 2008). One explanation is that, just as for Cascade, CSM is a surveillance complex that targets invading DNA and recruits a distinct nuclease to degrade targets. If so, the identity of this nuclease remains at present a matter for conjecture. Cas3 could in theory fulfill the role but is not always present in genomes harboring an active type IIIA system. The Csm6 protein is another possibility, although its structure bears more resemblance to families of transcription factors (Makarova et al., 2011b). It is conceivable that the nuclease varies in different lineages, which would be in keeping with the dynamic nature of the CRISPR system. Alternatively, the HD domain of the large subunit may be responsible for the degradation activity but be controlled in a manner that is not yet understood.

### Conclusions

This study has revealed clear similarities in the backbone structures of the CSM and Cascade surveillance complexes, suggesting a deep evolutionary relationship, as postulated from bioinformatics studies (Makarova et al., 2011a). Nonetheless, the differences should not be underestimated. For example, the requirement for a protospacer adjacent motif (PAM) in target sequences appears unique to the type I systems, and this may be reflected in the observation that the “large” subunits are not appreciably conserved between CSM and Cascade systems. Additional studies of the activity and mechanism of the CSM complex, both in vitro and in vivo, will be required in order to discern full details of role in the CRISPR system and its functional and structural relationship with Cascade.

## Experimental Procedures

### Expression and Purification of Tagged CSM Complex in *S. solfataricus*

The gene encoding the large subunit of the complex, *sso1428*, was amplified with oligonucleotides containing *Nco*I and BamHI restriction sites. Ligation of the restricted PCR product into pMZ1 (Zolghadr et al., 2007) yielded plasmid pMZ-1428. Expression from pMZ1 leads to the addition of a C-terminal tandem tag (Strep and 10× His) to the protein. The expression cassette was excised from plasmid pMZ-1428 and ligated into the virus-based expression vector pSVA9, yielding plasmid pSVA-1428, which was transformed into the *S. solfataricus* PH1-16 expression strain, as described previously (Albers et al., 2006). After transformation, cells were first cultivated in unselective Brock medium containing 0.2% tryptone and 10 μg/ml uracil, then transferred to selective media containing 0.2% glucose and NZ-Amine without uracil. Once the OD_600nm_ reached 0.6, cells were transferred to expression media containing 0.2% arabinose and NZ-amine to induce the expression of the tagged Sso1428 and then collected at an OD of 0.8–1.0. Later experiments involved the production of CSM complex tagged on subunit Sso1431 via the same methodology.

### Purification of Tagged CSM Complex from *S. solfataricus*

Cells were resuspended in buffer A (20 mM HEPES [pH 7.5], 250 mM NaCl, 30 mM imidazole) and disrupted by sonication for 6 × 3 min on ice. The lysate was centrifuged at 40,000 rpm for 45 min and loaded onto a Histrap column (GE Healthcare) equilibrated in buffer A. After being washed with 20 column volumes of buffer A, bound proteins were eluted with a linear gradient of buffer B (20 mM HEPES [pH 7.5], 250 mM NaCl, 1 M imidazole). Fractions containing the CSM complex were pooled, exchanged into buffer C (20 mM Tris·HCl [pH 8], 50 mM NaCl), and loaded onto a monoQ column (GE Healthcare) equilibrated with buffer D (20 mM Tris·HCl [pH 8], 50 mM NaCl, 1 mM EDTA, 1 mM DTT). Bound proteins were eluted with a linear gradient of buffer E (20 mM Tris·HCl [pH 8], 1 M NaCl, 1 mM EDTA, 1 mM DTT). Fractions containing the CSM complex were pooled, concentrated, and loaded onto a gel filtration column (S500, GE Healthcare) equilibrated with buffer F (20 mM Tris·HCl [pH 8], 150 mM NaCl). Fractions containing the CSM complex were pooled, concentrated, and stored at 4°C.

### Purification and Deep Sequencing of crRNA

RNA was extracted from the purified native CSM complex by the classical phenol/chloroform method followed by ethanol precipitation and vacuum desiccation. Dried RNA was resuspended in 5 μl of water and labeled in a 10 μl reaction containing polynucleotide kinase and 2 μCi γ^32^P-ATP. Labeled RNAs were analyzed by electrophoresis on a 15% acrylamide, 7M urea, Tris-borate-EDTA (TBE) denaturing gel and visualized by phosphorimaging. Small RNA libraries were prepared with the use of the Small RNA Sample Prep Kit according to the manufacturers’ instructions, starting from 100 ng RNA. The ligated RNA fragments were reverse transcribed, followed by ten cycles of PCR amplification. Subsequently, amplified libraries were purified on 6% polyacrylamide gels. The library was sequenced (36 bp single-read sequencing) with an Illumina Genome Analyzer IIx. Library preparation and sequencing was performed by the CNRS Imagif platform in Gif sur Yvette, France.This resulted in the addition of the adaptor sequence at the 3′ end of each sequence. Reads were processed, adaptor sequence was removed, and reads were mapped against the *S. solfataricus* P2 genome with the use of Galaxy (Blankenberg et al., 2010; Giardine et al., 2005; Goecks et al., 2010).

### Electron Microscopy

The CSM complex bound to crRNA was studied by negative-staining EM and single-particle analysis. Data were collected on an FEI F20 FEG microscope equipped with a 4k × 4k CCD camera. Images were collected under low-dose mode at a magnification of 29,000×, at a final sampling of 3.6 Å/pixel at the specimen level. Single-particle images were interactively selected with the Boxer program from the EMAN single-particle analysis package (Ludtke et al., 1999) and extracted into boxes. Image processing was performed with the IMAGIC-5 package (van Heel et al., 1996). The data set was resampled at 7.2 Å/pixel, and 7,829 images were band-pass filtered with a high pass cutoff of 110 Å and a low pass cutoff of 18 Å. The single-particle images were analyzed by multivariate statistical analysis with IMAGIC-5. The data set was subjected to successive rounds of alignment and classification in order to improve the resulting image class averages. We then generated a Gaussian blob, using the makeinitialmodel.py program from the EMAN package. The x, y, and z dimensions for the blob were chosen on the basis of the dimensions of class averages calculated with IMAGIC-5. Noise was added to the Gaussian blob with the use of the proc3d program in EMAN, to a 0.5 value. CSM class averages were aligned to the starting 3D volume by projection matching via the refine command in the EMAN package. The CMR/RNA structure was refined until the map converged. The resolution for the final reconstruction was calculated as ∼30 Å through the use of the 0.5 FSC criterion. To interpret the map, we fitted a portion of the EMD-5314 map (Wiedenheft et al., 2011). To obtain the core Cas7 backbone, we segmented EMD-5314 using the Segger routine in Chimera and generated a volume containing six proximal Cas7 domains. The seventh module within the backbone was the Cas5 subunit. Figures were prepared with UCSF Chimera (Goddard et al., 2007).

### Mass Spectrometry

#### Electrospray Ionization LC-MS Analysis of CSM Subunits

LC-MS analysis of individual CSM subunits was carried out on a Dionex Ultimate 3000 LC System (RSLCnano; Thermo) equipped with a 3 nl UV detector set at 214 and 280 nm. CSM was prepared in a 1:1 (v/v) mix of 0.1% TFA and 1 μl of sample applied to a PS-DVB reverse-phase monolithic column (Pepswift 100 μm i.d. × 25 cm; Thermo) equilibrated at 90% solvent A (0.05% TFA) and 10% solvent B (0.04% TFA, 90% ACN). A linear gradient of 10%–70% solvent B in 25 min at a flow rate of 600 nl/min was used. The column effluent was passed through a nanospray ionization interface into a QSTAR XL mass spectrometer (AB Sciex). For peptide analysis, the CSM complex was digested with tryspin (Promega). The resultant peptide mixture was separated on a reverse-phase C18 column (PepMap 75 μm i.d. × 50 cm; Thermo) before being analyzed on a LTO-Orbitrap XL hybrid mass spectrometer (Thermo). Eight proteins were identified as constituents of the CSM complex through a search against the NCBInr database using the Mascot search engine and are listed in Table S2.

#### Relative Quantification of CSM Subunits

For quantification of the relative amount of each individual CSM subunits, the complete inventory of CSM tryptic peptides was surveyed. One or two peptides per subunit were selected for quantification according to previously published criteria (Schmidt et al., 2010). A library of synthetic dipeptides was then ordered from Thermo, containing each of these selected peptides combined with the sequence of a reference peptide (GSVDLNYLR) of subunit Sso1428. The dipeptides were isotopically labeled with (^15^N;^13^C) R/K residues to give a theoretical molar ratio of 1:1 and a mass increase (10/8 Da for R/K residues, respectively) for the component monopeptide upon trypsin cleavage. Subsequently, an aliquot of CSM complex was spiked with each of the synthetic dipeptide and the mixture was subjected to trypsin cleavage. The resulting digests were surveyed on the LTQ-Orbitrap. The extracted total ion chromatograms for the light and heavy peptide pairs were compared and their relative ratios calculated as quotients of the plotted peak areas.

#### Chemical Crosslinking of CSM Subunits Analyzed by MS

The crosslinking experiment was initiated by mixing 2 μl of a 1:1 mixture of 12.5 mM deuterated (d4) and 12.5 mM nondeuterated (d0) BS3 crosslinkers with 20 μl aliquot of CSM complex at a concentration of 1 μg/μl. The reaction mixture was incubated for 1 hr at room temperature, and a control was prepared for comparison without addition of the crosslinkers. Potential crosslinked peptides were identified through the use of the MassMatrix Database Search Engine (Xu et al., 2008a; Xu et al., 2008b) and manually validated by (1) checking the presence of parent d4/d0 ion pairs in the MS spectra, (2) checking their absence in the control, and (3) checking qualities of the corresponding tandem MS spectra.

#### MS and IM-MS of the CSM Complex and Subcomplexes

For MS of the intact complex, 20 μl of purified CSM (6 μg/μl) was exchanged into 200 mM AmAc buffer (pH 7.5) with the use of Micro Bio-Spin 6 Columns (Bio-Rad). The sample was diluted 1:10 into AmAc buffer, and 2 μl aliquots were electrosprayed from gold-coated borosilicate capillaries prepared in house. Spectra were recorded on a QSTAR XL (AB Sciex) modified for high mass detection (Sobott et al., 2002) and adjusted for the preservation of noncovalent interactions (Hernández and Robinson, 2007). MS experiments were performed at a capillary voltage of 1,200 V and declustering potentials of 40 V and 15 V. In tandem MS experiments, ions were isolated in the quadrupole and subjected to collision-induced dissociation (acceleration energy up to 200 V). For subcomplex generation, a 0.5 μl aliquot of the CSM solution was mixed with 19.5 μl of 200 mM AmAc containing incremental concentrations of acetic acid (5%–20% v/v) immediately before MS analysis.

All IM-MS spectra were recorded on a hybrid quadrupole (Q)-IM-ToF MS instrument known as Synapt G2 HDMS (Giles et al., 2011) and incorporating traveling-wave ion guide for IM separation (Waters). The instrument is modified for high mass transmission (Sobott et al., 2002) and uses nitrogen for mobility separation with the trap and transfer regions filled with argon. The Synapt G2 was operated at 3.21 mbar and 3.80 × 10^−2^ mbar for mobility and trap/transfer regions, respectively, which are separated by a "helium gate" pressurized at 1.41 bar. Ions were injected into the mobility cell at a 100 μs pulse with an injection voltage of 15 V. IM measurement for the CSM complex and subcomplexes was performed in triplicate, employing different combinations of wave height (WH) and wave velocity (WV) as follows: WH = 32V and WV = 800ms^−1^; WH = 32V and WV = 700ms^−1^; WH = 30V and WV = 700ms^−1^.

#### Coarse-Grain Modeling of CSM

An iterative series of modeling steps was employed for the CSM modeling combining information from MS and IM-MS, chemical crosslinking, and quantification experiments. First, each subunit (but the subunit of Sso1428 was divided into two domains) was represented as a sphere with a radius derived from its corresponding mass. We then employed a Monte Carlo sampling approach to build a large number of structures (10,000 models) for the CSM complex and subcomplexes consistent with the input connectivity data from MS-based experiments. Next, all generated models were scored and subsequently ranked on the basis of the violation of calculated CCSs values of model structures to the experimental values measured by IM. Finally, the top-scoring models were fitted into the EM map and the model with the best fit was selected as the final solution.

## Figures and Tables

**Table 1:** Quantification of CSM Subunits Relative to the Largest Subunit Sso1428

Subunits To Be Quantified	Selected Peptides	Ratio of Unknown Subunit:Sso1428				
		Repeat 1	Repeat 2	Repeat 3	Average	STD
Sso1432	^18^VGGGQEVGDNVIR^30^	0.92	0.91	0.96	0.93	0.03
Sso1431	^293^ISDLSSILNK^302^	0.67	0.65	0.70	0.67	0.03
Sso1430	^150^LLLYSILDLR^159^	0.81	0.76	0.83	0.80	0.04
Sso1430	^199^YLWEAENK^206^	1.12	1.09	1.15	1.12	0.03
Sso1426	^136^FLDSLPISYSLNTR^149^	4.85	4.81	4.73	4.80	0.06
Sso1425	^62^SLVESYTK^69^	1.45	1.35	1.56	1.45	0.11
Sso1427	^129^IFNPDPNR^136^	0.80	0.79	0.83	0.81	0.02
Sso1424	^1^N-acetyl-sSQDLLDIATR^11^	3.62	3.51	4.03	3.72	0.27

**Table 2:** Collision Cross Sections of CSM Complex and Subcomplexes Measured by IM-MS

CSM (Sub-) Complexes	Mass (kDa)	Experimental CCS (nm^2^)				Calculated CCS (CG Model)	Difference (%)
		WH=32V WV=800s^−1^	WH=32V WV=700s^−1^	WH=30V WV=700s^−1^	Average		
Intact	427	170.3	168.6	172.9	170.6	171.1	+0.3
Subcomplex I	357	146.6	146.0	147.1	146.6	146.4	−0.1
Subcomplex II	216	101.6	98.9	101.1	100.5	97.6	−2.9
Sso1428	97	55.1	55.0	56.5	55.6	56.1	+0.9

CCS, collision cross sections.

## Accession Numbers

The raw sequence data have been uploaded to the Sequence Read Archive under accession number ERP003555. The CSM EM map has been submitted to the EMDB under accession number EMD-2420.
