# Supplementary material for: Structure of the CRISPR Interference Complex CSM Reveals Key Similarities with Cascade
Source: Mol Cell. 2013 Oct 10;52(1):124–34. doi: 10.1016/j.molcel.2013.08.020 (PMC3807668; doi:10.1016/j.molcel.2013.08.020)
Supplement: Document S1. Tables S1–S6 and Figures S1–S4 [file mmc1.pdf]

Molecular Cell, Volume *52*

Supplemental Information

Structure of the CRISPR Interference Complex CSM Reveals Key Similarities with Cascade

Christophe Rouillon, Min Zhou, Jing Zhang, Argyris Politis, Victoria Beilsten, Giuseppe Cannone, Shirley Graham, Carol V. Robinson, Laura Spagnolo, and Malcolm F. White

Table S1. Distribution of CSM-Bound crRNA Matches to the *S. solfataricus* P1 CRISPR Loci. (related with Figure 1)

**CRISPR Locus**	**A**	**B**	**C**	**D**	**E**	**F**
Size of locus (nt)	5677	3943	2124	11334	410	6033
RNA matches	3060221	1805666	204202	327035	139	52613
% of total matches	56.1	33.1	3.75	6.0	0.003	0.97
Density (matches per nt)	539	458	96	29	0.3	9

Table S2. Measured Subunit Masses and Post-translational Modifications Identified for the CSM Complex (related with figure 3)

**Uniprot ID**	**Subunit Name**	**Post-translational modifications**	**Expected Mw (Da)**	**Measured Mw (Da)[a]**	**Measured Mw (Da)[b]**
**Q97YA8**	Sso1424	Loss of first 15 residues; Acetylation Ser-1; Methylation R76,K85,K92,R94,K130,K138	15,948	14,082	14,082
**Q97YA2**	Sso1430	Methylation K97, K181, K184, K206	23,200	23,226	23,225
**Q97YA7**	Sso1425	Methylation K95, K241, Phosphorylation T138	27,939	27,799	27,619
**Q97YA0**	Sso1432	Methylation K47, R51, K69, K70, K82	28,917	28,846	28,799
**Q97YA6**	Sso1426	Methylation K247, K265, K275, K280/281	31,527	31,040	31,028
**Q97YA5**	Sso1427	Methylation K65/66, K86, K176, K192, K203/206, K221, K257, K264	30,939	31,086	31,095
**Q97YA1**	Sso1431	Methylation K9, K93, K130, K215, K231/235, Phosphorylation S227, T232	34,895	37,411	34,914
**Q97YA4/3**	Sso1428	Methylation K39, K110, K118/119, K204, K217, R222, K256, K263, K298/R299, K312, K443, K687/693, K712, K720, K722, K749, R761/K762, K804, K812/R814	94,757	95,005	97,567

CSM purified with 10×His-tag attached to the subunit Sso1431[a] and Sso1428[b]

Table S3. Synthetic Labelled Di-peptides for Quantification (related with figure 3)

Labelled Di-peptides	labelled residues	Reference subunit	Target subunit
^{1}GSVDLNYLRVGGGQEVGDNVIR^{22}	R9;R22	Sso1428	Sso1432
^{1}GSVDLNYLRISDLSSILNK^{19}	R9;K19	Sso1428	Sso1431
^{1}GSVDLNYLRLLLYSILDLR^{19}	R9;R19	Sso1428	Sso1430
^{1}GSVDLNYLRYLWEAENK^{17}	R9;R17	Sso1428	Sso1430
^{1}GSVDLNYLRFLDSLPISYSLNTR^{23}	R9;R23	Sso1428	Sso1426
^{1}GSVDLNYLRSLVESYTK^{17}	R9;K17	Sso1428	Sso1425
^{1}GSVDLNYLRIFNPDPNR^{17}	R9;R17	Sso1428	Sso1427
N-acetyl-sSQDLLDIATRGSVDLNYLR^{19}	R10;R19	Sso1428	Sso1424

Table S4. List of Hits (within ±10% error) Searching for Mass Measured for the Intact CSM Complex (427,789Da) (related with Table 1)

Sso 1424	Sso 1430	Sso 1425	Sso 1432	Sso 1426	Sso 1427	Sso 1431	Sso 1428	crRNA	Expected mass (Da)	Error (%)
**3**	**1**	**1**	**1**	**4**	**1**	**1**	**1**	**1**	**426,374**	**-0.3%**
4	1	1	1	4	1	1	1	1	440,458	+3.0%
3	1	2	1	4	1	1	1	1	454,173	+6.2%
3	1	1	1	5	1	1	1	1	457,414	+6.9%
4	1	2	1	4	1	1	1	1	468,257	+9.5%

Table S5. Experimentally Determined Masses of CSM Complex and Subcomplexes[a] (related with figure 3)

CSM complexes	Expected Mass (Da)	Experimental Mass (Da)	Error (%)
Intact: $(Sso1424)_3$ crRNA Sso1430 Sso1425 Sso1432 $(Sso1426)_4$ Sso1427 Sso1431 Sso1428	426,374	427,789[a]	0.3
– Sso1424	412,458	413,752	0.3
– Sso1428	328,742	329,828	0.3
– Sso1426	395,502	396,778	0.3
– $(Sso1424)_2$ Sso1425	370,575	371,437	0.2
– $(Sso1424)_2$ Sso1425 Sso1432	341,775	342,358	0.2
– $(Sso1424)_3$ Sso1425 Sso1432	327,691	328,396	0.2
– $(Sso1424)_3$ Sso1425	356,491	357,318	0.2
– $(Sso1424)_3$ Sso1425 Sso1426	325,451	326,201	0.2
+ Sso1430 Sso1426 Sso1427 Sso1431 Sso1428	217,751	217,862	0.1
+ Sso1430 Sso1427 Sso1431 Sso1428	186,711	186,826	0.1
+ Sso1426 Sso1427 Sso1431 Sso1428	194,525	194,722	0.1
+ Sso1430 Sso1431 Sso1428	155,621	155,784	0.1
+ Sso1427 Sso1431 Sso1428	163,485	162,665	0.1
+ Sso1430 Sso1428	120,726	120,804	0.1
+ $(Sso1424)_2$	28,168	28,199	0.1
+ Sso1424 Sso1425	41,883	41,935	0.1
+ Sso1424 Sso1427	45,174	45,230	0.1
+ Sso1426 Sso1426/7	62,130	62,201	0.1
+ Sso1425 Sso1432	56,599	56,669	0.1

[a]Mass measured for the intact CSM purified by tagging the subunit Sso1428

Table S6. Inter-subunit Contacts Established for CSM *via* Chemical Crosslinking (related with figure 3)

Crosslinks	Charge	m/z	MH^+ (monoisotopic)	Sequences	Subunits[a]
1	5<br>4	428.61<br>535.51	2139.03	EAHK($1)NGVCDVCK<br>MFK($1)R	1430(71)-1429(108)
2	3<br>4	743.09<br>557.57	2227.25	SLVESYTK($1)SLNDSK<br>VIK($1)R	1425(72)-1426(16)
3	4<br>3	360.96<br>480.94	1440.80	SDLK($1)NYR K($1)EK	1429(175)-1431(91)
4	4<br>3	490.80<br>652.72	1960.17	LLLASLK($1)DR<br>SVSDVK($1)R	1424(92)-1427(53)
5	4	387.99	1548.93	AK($1)SVEALK<br>K($1)ASEK	1425(187)-1432(168)
6	3	690.70	2070.09	SLVESYTK($1)SLNDSK<br>K($1)SK	1425(72)-1432(165)
7	3	603.35	1808.05	EDIK($1)FKLDK<br>DK($1)IR	1428(528)-1429(226)
8	3	550.65	1649.94	SEENSLIK($1)R<br>TGK($1)K	1429(263)-1428(371)

[a]Sso1428 and Sso1429 were searched and represented separately.

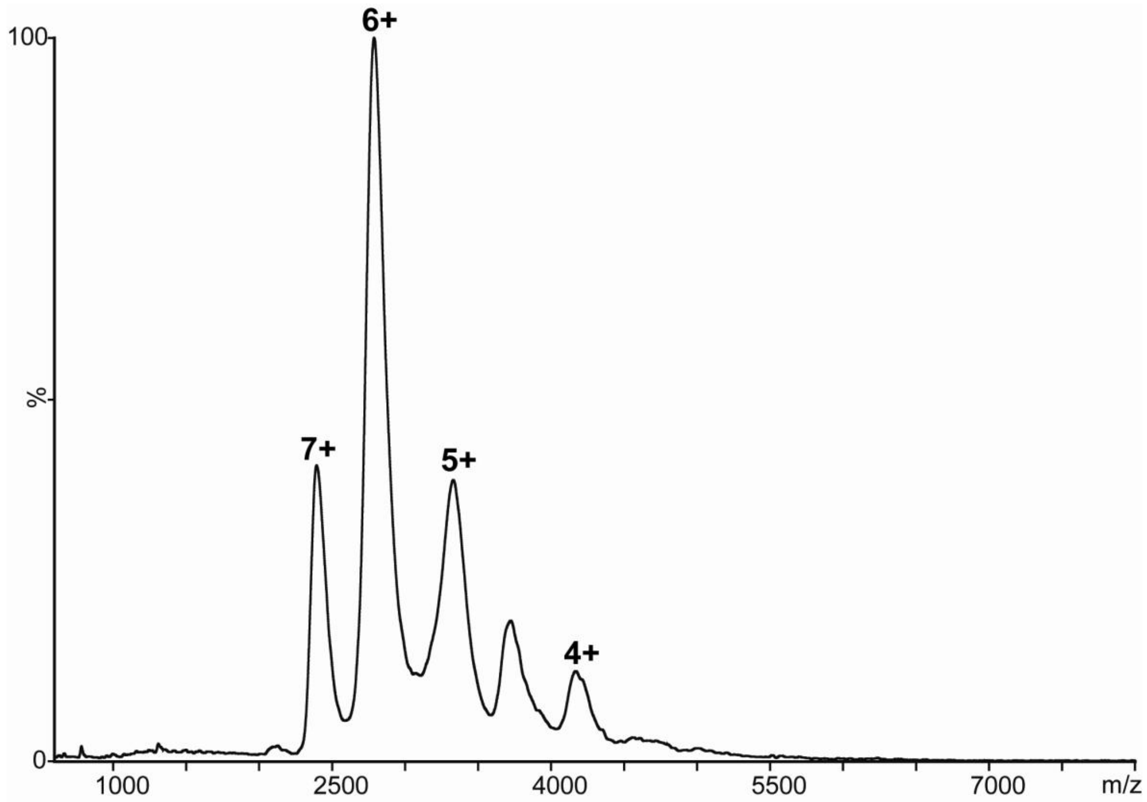

Figure S1 MS spectrum recorded for crRNA molecules extracted from the CSM complex. The spectrum shows a single charge state series with a mass centred around 16,520 Da. The broad charge state peaks are consistent with the sequence heterogeneity of the crRNA. (related with figure 3)

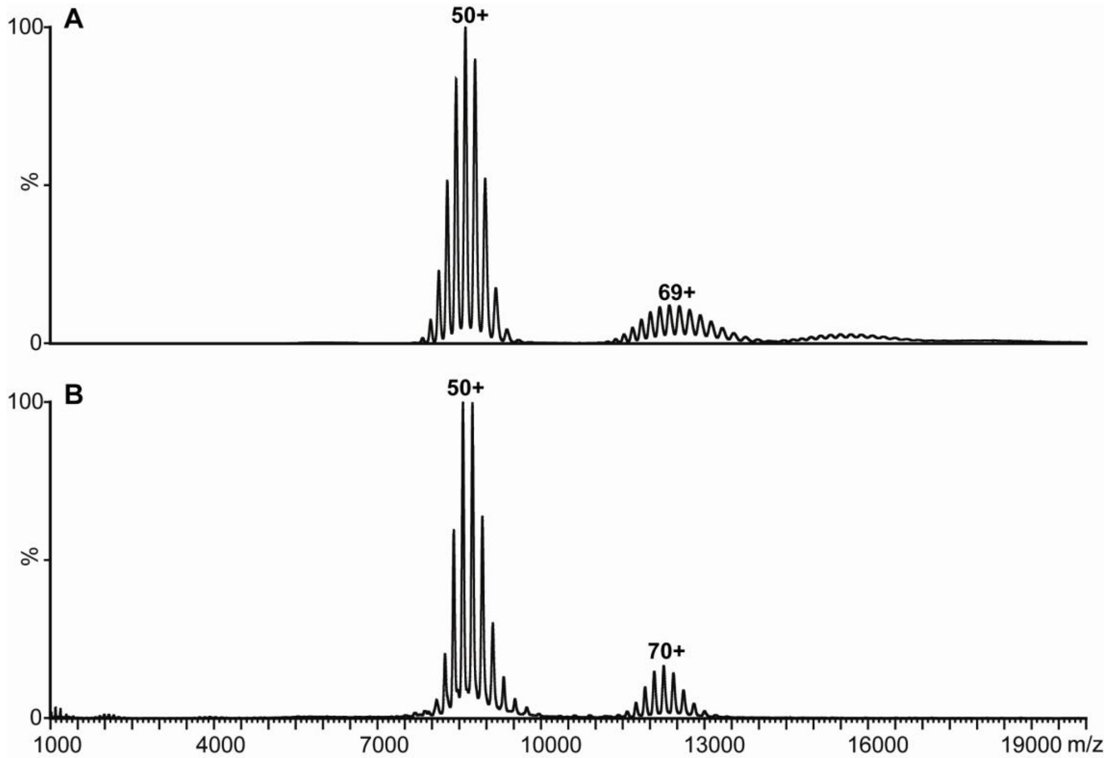

Figure S2 MS spectra recorded for the intact CSM complex purified with a 10×His-tag attached to the subunit Sso1431 (A) or Sso1428 (B). Both spectra show a single well resolved charge state series with a measured mass of 427,611 Da (A) or 427,789 Da (B), suggesting a homologous complex with the subunits Sso1431 and 1428 present in stoichiometric amounts. (related with figure 3)

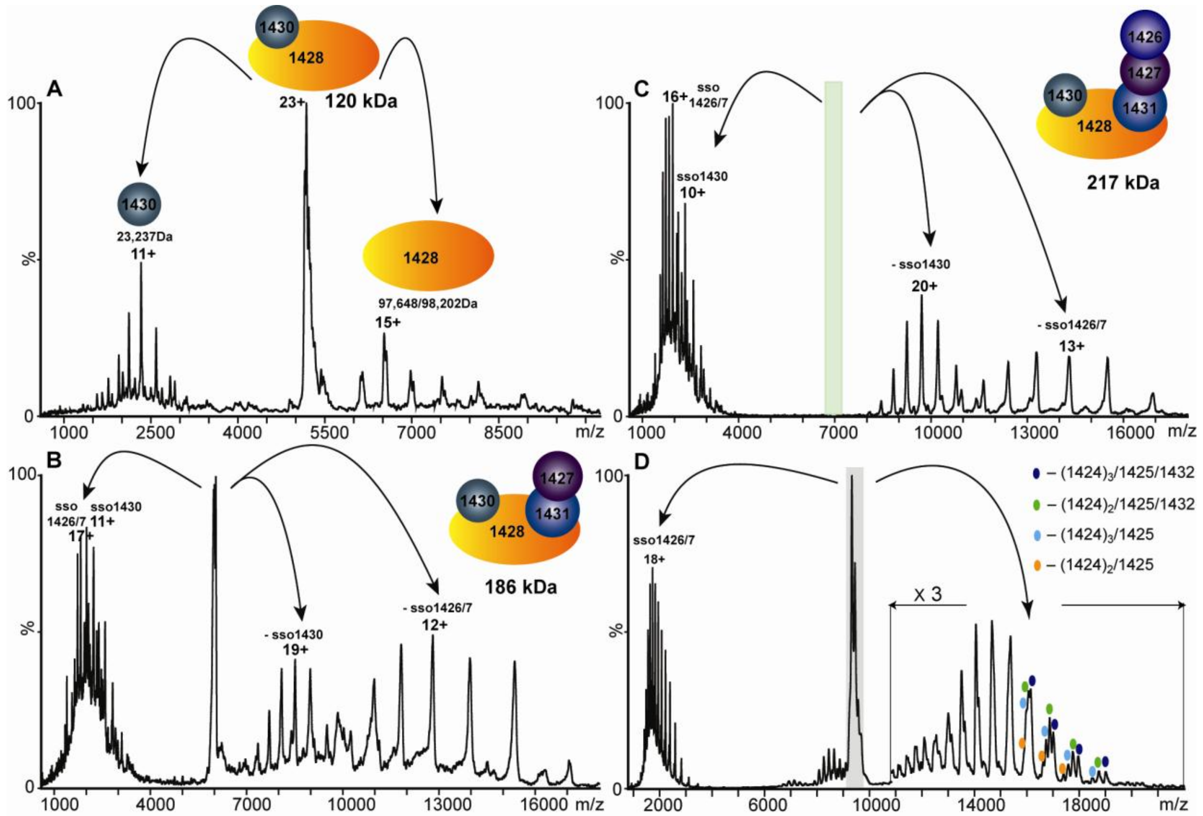

Figure S3 Tandem MS spectra of CSM subcomplexes CID experiments of the 120 kDa (A), 186 kDa (B) and 217 kDa (C) subcomplexes enabled assignment of the "core" of CSM comprising single copies of sso1428, 1430, 1431 and two copies of 1426/7. A mixed selection of parent ions in (D) lead to split CID peaks, and were assigned to sub-complexes having lost different combinations of the subunit sso1424, 1425 and 1432.

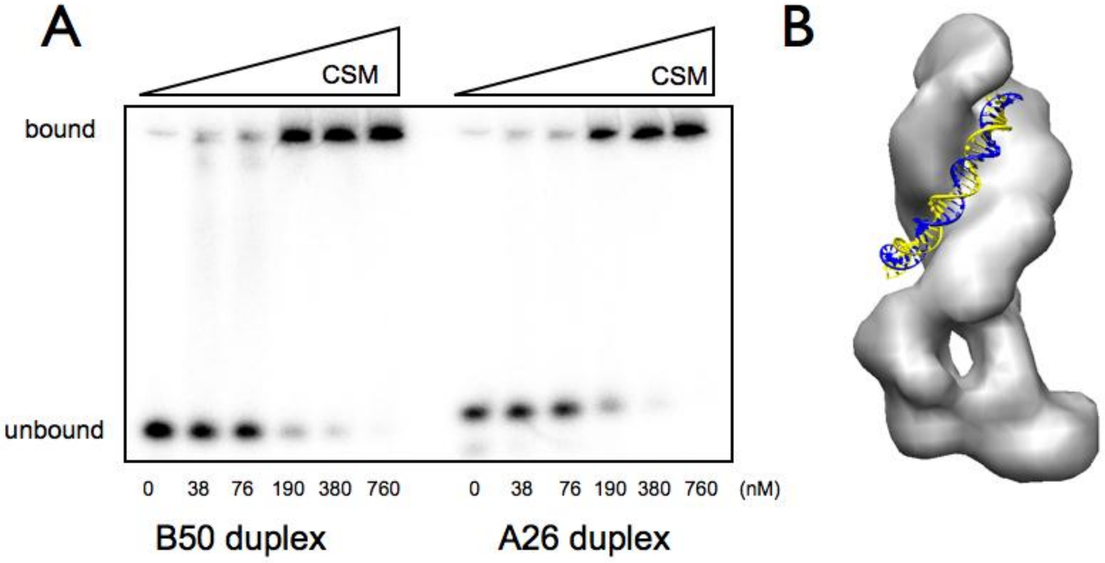

A26 duplex: 5′-GATCCAGCCA**ACCATACCCAACTTCTAACAACGTCGTTCTTAACAACG**TGGAGAG

B50 duplex: 5′-CCTCGAGGGATCCGTCCTAGCAAGCCGCTGCTACCGGAAGCTTCTGGACC

Figure S4. Double-stranded DNA binding by the purified CSM complex

A. The CSM complex was incubated with labelled duplex DNA species (corresponding to the abundant spacer A26 or a control sequence B50) and DNA binding was assessed by gel electrophoretic retardation. The complex shifted both species with high affinity and an apparent dissociation constant around 100 nM. This suggests that CSM possesses a dsDNA binding site, consistent with its proposed function. Sequence A26 corresponds to an abundant crRNA sequence (Locus A spacer 26) identified in the CSM complex by deep sequencing, but it only constitutes < 5% of the total crRNA bound in the complex. This probably explains the failure to observe sequence specific DNA binding by the CSM complex. The A26 spacer sequence is shown in bold text.

B. Model showing a 38 bp DNA duplex in complex with the CSM protein. The DNA was fitted manually to show the potential binding site along the Cas7 backbone that is thought to bind the crRNA. (related with figure 5)
